# Neural Sensitivity to Conversational Inter‐Speaker Gaps in the Broad Autism Phenotype

**DOI:** 10.1111/psyp.70355

**Published:** 2026-07-03

**Authors:** Wei Siong Neo, Kamryn M. Witkowiak, Felicia Roberts, Bridgette Kelleher, Mai Yamamoto, Dan Foti

**Affiliations:** ^1^ Department of Psychological Sciences Purdue University West Lafayette Indiana USA; ^2^ Brian Lamb School of Communication Purdue University West Lafayette Indiana USA; ^3^ Department of Linguistics Purdue University West Lafayette Indiana USA

**Keywords:** broad autism phenotype, event‐related potential, inter‐speaker gap, P3, pragmatic language, stimulus‐preceding negativity

## Abstract

Pragmatic language differences are present in the broad autism phenotype (BAP). Objective markers of pragmatic language subprocesses, such as inter‐speaker turn‐taking, may enhance understanding of these differences. Here, we examined how neural and behavioral responses elicited by inter‐speaker turn‐taking are related to the BAP, using well‐validated conversational stimuli with different inter‐speaker gap durations in a stratified sample of 63 adults selected for varied autism‐associated traits. As outcomes, we measured ERPs elicited by inter‐speaker gap (stimulus‐preceding negativity) and speaker response (P3), and subjective ratings of conversation quality. Among individuals with more autism‐associated traits, extended inter‐speaker gaps were considered less salient as indicated by reduced P3. However, monitoring of inter‐speaker gaps and perceived conversation quality were unrelated to autism‐associated traits. These findings provide preliminary evidence that pragmatic inference of inter‐speaker gaps may be specifically disrupted in those endorsing a higher degree of autism‐associated traits and detectable with the P3 ERP, laying the foundation for similar neurobehavioral studies in autism.

## Impact Statement

Pragmatic language differences exist in the broader autism phenotype. In the first known study to examine how neural and behavioral responses elicited by conversational inter‐speaker gaps may be affected in the broad autism phenotype, we present initial evidence that pragmatic inference of inter‐speaker gaps may be specifically disrupted. This is evidenced by individuals with more autism‐associated traits perceiving uncharacteristically extended inter‐speaker gaps as less salient.

## 1 Introduction

Autism spectrum disorder (ASD) is a neurodevelopmental condition characterized by persistent differences in social communication and interactions (American Psychiatric Association [APA] 2022). Despite substantial heterogeneity in language profiles across individuals (Wilkinson 1998), pragmatic language differences have been consistently reported and considered to be a hallmark feature of ASD (Friedman and Sterling 2019; Groen et al. 2008). These challenges in the social aspects of language often occur during conversations and other discourse contexts, manifesting as a variety of differences, including difficulties in drawing inferences from narratives, issues with appropriate conversational turn‐taking, limited understanding of figurative language, and reduced abilities in using contextual information to resolve ambiguities (Andrés‐Roqueta and Katsos 2017). Notably, autistic adults have expressed how their communication differences have far‐reaching consequences on their lives (e.g., feelings of vulnerability and withdrawal from society; Cummins et al. 2020) and caregivers of autistic individuals frequently cite conversational skills as a key outcome priority (Knott et al. 2006), highlighting the functional significance of pragmatic language differences to autistic individuals and their families.

Consistent with the current dimensional conceptualization of ASD (APA 2022), the behavioral, cognitive, and social characteristics that define ASD may be present in neurotypical individuals to a lesser degree and at varying levels (Sarovic 2021). These subclinical traits that are milder expressions of the defining characteristics of ASD constitute the broad autism phenotype (BAP; Ingersoll and Wainer 2014). Perspectives on the BAP have shifted over time, with initial theoretical conceptualizations emphasizing its increased prevalence in biological relatives of autistic individuals; more recent conceptualizations consider BAP features as being continuously distributed in the general population (Wainer et al. 2011). Indeed, the BAP may be construed as a personality inventory given its normal distributional patterns and temporal stability (Sarovic 2021). Critically, parallel to findings in ASD, pragmatic language differences and reduced sensitivity to nonverbal cues are associated with the BAP in the general population (Ingersoll 2010; Ingersoll and Wainer 2014). For example, undergraduate students with more BAP characteristics exhibited poorer social skills during a live conversational task (Sasson et al. 2013), raising the possibility that common processes may underlie social communication challenges in the BAP and ASD. As such, examining pragmatic language differences in the BAP may offer complementary insights on disrupted social language processes in ASD and advance clinical assessments and interventions of pragmatic language.

Assessing pragmatic language abilities remains challenging, despite the development of various formal assessments, rating scales, and structured observations (for a list of widely used measures, see Norbury 2014). These difficulties partially stem from the need to consider multiple factors beyond conversational content and features (e.g., cultural rules and social dynamics) as well as limited normative data on pragmatic skills (e.g., appropriate duration of eye contact with a conversational partner). One approach to address these challenges is to leverage psychophysiological methodologies to focus on specific aspects of social language behaviors that are well characterized and amenable to systematic, empirical investigation. Measuring individuals' responses to conversational stimuli in real time may also provide insights on specific neural processes that contribute to pragmatic language differences between autistic and neurotypical individuals. From a translational perspective, identifying these distinct neural processes may yield more personalized, process‐based interventions of conversational skills.

In the present study, we focused on two key subprocesses that occur during processing of conversational inter‐speaker gaps, that is, periods of silence between speaker turns during conversations, which convey considerable pragmatic information and are fundamental for understanding conversational systems (Levinson and Torreira 2015): (a) monitoring of turns (i.e., keeping track of the transition from the end of a speaker's utterance to the initiation of the other speaker's utterance); and (b) pragmatic inference on the timing of speakers' utterances (i.e., considering possible unspoken meanings conveyed by the duration of inter‐speaker gaps when interpreting a speaker's utterance). Importantly, disrupted processing of inter‐speaker gaps may manifest as conversational differences in ASD (e.g., frequent interruptions, limited conversational balance, and reduced perspective‐taking) and the BAP (e.g., delayed relinquishing of conversational turns and misinterpretation of speakers' statements).

Event‐related potentials (ERPs) offer high temporal precision to map out neural processes in real time and are thus well suited for studying how individuals process conversational inter‐speaker gaps, which are typically on the order of milliseconds to seconds (Kendrick and Torreira 2015; Roberts and Francis 2013; Stivers et al. 2009). Moreover, ERPs provide a means to investigate processes that occur during inter‐speaker gaps, which may be challenging to observe behaviorally. For example, Foti and Roberts (2016) examined ERPs elicited during two stages of conversation processing in an auditory task explicitly designed to focus on the biobehavioral effects of different inter‐speaker gap durations. They employed a series of auditory stimuli that simulated phone conversations between two friends that ended with the recipient agreeing to a request by the caller, either after an appropriate pause of 200 ms or after an extended pause of 700 ms. Several key biobehavioral findings emerged from their manipulation of inter‐speaker gap duration. First, the stimulus‐preceding negativity (SPN), an ERP that reflects stimulus anticipation (Brunia et al. 2012), was present during inter‐speaker gaps, indicating that participants were expecting and monitoring for recipients' responses to the requests made by callers. Second, given that participants were asked to rate recipients' responses, those responses elicited the P3 complex, an ERP that indexes the allocation of attention to task‐relevant stimuli (Polich 2012). Given that the P3 complex is also modulated by violations of expectancy (Donchin 1981), affirmative responses by recipients after extended pauses yielded larger P3 complexes than those after appropriate pauses, indicating that participants were not expecting recipients to provide affirmative responses after extended pauses and perceived them as anomalous. P3 amplitude is known to be strongly influenced by a combination of stimulus probability, stimulus intensity, and inter‐stimulus interval (Polich 1990). In this task, normal and long inter‐speaker gaps are equally probable; thus, P3 modulation likely reflects a combination of inter‐stimulus interval and expectancy violation. Finally, consistent with findings from behavioral studies on inter‐speaker gaps (Kendrick and Torreira 2015; Roberts and Francis 2013), recipients' responses were rated more negatively when they occurred after extended pauses than after appropriate pauses, suggesting that participants considered recipients who agreed to requests after extended pauses to have been more reluctant.

The present study expands on this foundational work by examining whether conversational processes identified by Foti and Roberts (2016) in an unselected adult sample present atypically in the BAP. We had three study objectives. First, given limited neuroscience research on conversational inter‐speaker gaps, we aimed to replicate the biobehavioral effects of inter‐speaker gap duration in a novel, nonclinical sample of adults selected to differ in their degree of BAP features. Specifically, we expected to replicate within‐subject findings that: (1a) long inter‐speaker gaps would elicit a sustained SPN between requests and responses; and affirmative responses to requests after long inter‐speaker gaps would (1b) elicit an increased P3 complex and (1c) be rated more negatively than those after normal inter‐speaker gaps.

Second, despite strong support for pragmatic language differences in the BAP, it remains unclear whether processing of conversational inter‐speaker gaps is disrupted in the BAP, and if so, which subprocesses may be specifically affected. To address this literature gap, we examined how three biobehavioral effects of inter‐speaker gap duration might be related to the BAP. Specifically, we investigated between‐subjects, bivariate relationships between the degree of autism‐associated traits and (2a) SPN elicited by long inter‐speaker gaps; (2b) difference in P3 complexes elicited by long and normal inter‐speaker gaps; and (2c) difference in ratings elicited by long and normal inter‐speaker gaps. We broadly predicted that individuals with more autism‐associated traits would be less sensitive to distinctions between long and normal inter‐speaker gaps.

Finally, given that the BAP consists of several phenotypic characteristics, we conducted exploratory analyses as a first step toward probing specific BAP features that might be driving predicted associations between the BAP and biobehavioral effects of inter‐speaker gap duration. We tentatively hypothesized that BAP characteristics with more direct relevance to social communication (e.g., pragmatic language) would exhibit stronger associations with biobehavioral effects than those with less direct relevance (e.g., rigid personality).

## 2 Method

### 2.1 Participants

Sixty‐three undergraduate and graduate students from Purdue University participated in this study. Table 1 presents demographic details. We recruited the largest possible sample within budgetary constraints; with *N* = 63, this study had 80% statistical power to detect medium effect sizes, including *d* ≥ 0.36 and *r* ≥ 0.34. Inclusion criteria included age between 18 and 40 years, fluency in English, no history of any neurological illnesses, and no history of severe head trauma with loss of consciousness. Notably, we used a prescreening questionnaire that included a subset of questions from the Broad Autism Phenotype Questionnaire (BAPQ; Hurley et al. 2007)^1^ to facilitate oversampling of participants with relatively low and high behavioral traits associated with autism. Specifically, we oversampled participants with low and high scores on the subset of BAPQ questions, defined as 1.5 standard deviations below and above the mean BAPQ score, respectively (Ingersoll et al. 2011). Of the 63 study participants, 3 and 19 participants received low and high BAPQ scores, respectively, on the prescreening questionnaire. While we used a categorical approach (i.e., low and high BAPQ scores) during recruitment to maximize variability in the BAP, BAPQ total and subscale scores were treated as dimensional for analytic purposes. The local institutional review board approved this study. All participants provided written informed consent and received $60 for their participation.

**TABLE 1:** Participant demographics.

Characteristic	*M*	SD	Range
Age	22.14	3.74	18–34
	***n***	**%**	
Sex			
Female	32	50.8	
Male	31	49.2	
Race/Ethnicity			
Asian	15	23.8	
Biracial/Multiracial	5	7.9	
Black	1	1.6	
Hispanic/Latino	2	3.2	
White	40	63.5	

### 2.2 Procedure

This study consisted of an online prescreening questionnaire and an in‐person laboratory visit. Students enrolled at Purdue University received an email invitation to complete the prescreening questionnaire that contained a subset of BAPQ questions, if they were interested in participating in this study. During the laboratory visit, participants completed the full BAPQ,^2^ a cognitive assessment, and experimental tasks while electroencephalography (EEG) signals were recorded, as detailed below.

### 2.3 Broad Autism Phenotype Measure

The BAPQ (Hurley et al. 2007) is a 36‐item self‐report questionnaire that assesses language and personality characteristics postulated to be defining features of the BAP. Individual items are rated on a 6‐point scale, anchored by *very rarely* (1) and *very often* (6). The scores of all 36 individual items are averaged to produce a total score, with higher scores indicating more subclinical traits associated with autism. The BAPQ also yields scores for three subscales: *aloof personality*, *pragmatic language problems*, and *rigid personality*. Excellent internal consistency was established during scale development involving parents of autistic individuals or typically developing children (Hurley et al. 2007).

### 2.4 Cognitive Assessment Measure

The National Institutes of Health Toolbox—Cognition Battery (NIHTB‐CB) is a computerized assessment tool that contains seven subtests designed to assess multiple cognitive constructs, including attention, episodic memory, executive functioning, language, processing speed, and working memory (Weintraub et al. 2013). Of particular relevance are two language‐focused subtests (i.e., Oral Reading Recognition Test and Picture Vocabulary Test; Gershon et al. 2014). For the Oral Reading Recognition Test that measures reading decoding abilities, participants are shown a word in each trial and asked to read it aloud. For the Picture Vocabulary Test that measures receptive vocabulary, participants are shown four images in each trial and asked to select the image that best matches the meaning of an aurally presented word. Each subtest yields an age‐corrected standard score with a normative mean of 100 and standard deviation of 15. Additionally, all seven subtests may be combined to form a total composite score with a normative mean of 100 and standard deviation of 15, which represents overall cognitive functioning. Both language‐focused subtests and the total composite score demonstrated good test–retest reliability and strong convergent validity with gold‐standard cognitive measures (Gershon et al. 2014; Heaton et al. 2014). We included the NIHTB‐CB in this study to examine whether biobehavioral effects of inter‐speaker gap duration in the BAP reflect specific challenges with processing pragmatic features in conversations, rather than broad differences in cognitive functioning or language processing.

### 2.5 Conversation Processing

This study focused on an auditory task involving short conversation stimuli (for details, see Foti and Roberts 2016). Briefly, the task consisted of 28 trials (i.e., 20 target trials and 8 distractor trials) that were presented in two blocks with an intervening self‐paced break using Presentation (Neurobehavioral Systems 2019). On each trial, participants were presented with a telephone conversation between two friends that lasted approximately 10 s. All conversation stimuli involved mundane themes, with initial greetings followed by the caller making a simple report (e.g., “The callout flyers are ready.”). For target trials, the conversation continued with the recipient briefly acknowledging the caller's statement, followed by the caller making a related request (e.g., “Can you give me a ride over there?”), to which the recipient always provided an affirmative “sure” response; for distractor trials, the conversation continued with the recipient briefly responding to the caller's statement.^3^ Notably, we manipulated inter‐speaker gap duration on target trials, that is, the period of silence between the caller's request and the recipient's affirmative response. Specifically, inter‐speaker gap duration was 200 ms on half of the trials (i.e., trials with normal inter‐speaker gaps) and 700 ms on the remaining half of the trials (i.e., trials with long inter‐speaker gaps). These inter‐speaker gap durations were selected based on descriptive analyses and experimental findings of conversational turn‐taking—affirmative responses typically occur within 200 ms of requests in naturalistic conversations and delayed affirmative responses occurring beyond 700 ms of requests in experimental tasks are perceived as more reluctant or anomalous, given normative expectations of rejections for such delayed responses (Kendrick and Torreira 2015; Roberts and Francis 2013; Stivers et al. 2009). Critically, the affirmative “sure” responses were identical across all target trials. In other words, the acoustic properties of affirmative responses for both trials with long and normal inter‐speaker gaps were kept constant, such that any biobehavioral effects of inter‐speaker gap duration could not be attributed to variations in vocal qualities of affirmative responses. At the end of each trial, participants rated an affective characteristic of the recipient's affirmative response to the caller's request (e.g., degree of interest or willingness) on a 6‐point scale, with higher ratings representing more positive affective perceptions.

### 2.6 Electrophysiological Recording and Processing

Participants' EEG data were continuously recorded using the BrainVision actiCHamp 24‐bit amplifier and 32 actiCAP active electrodes (Brain Products 2022). EEG signals were sampled at 500 Hz through BrainVision PyCorder (Brain Products 2015) without any online filters. Additionally, participants' electrooculography data were continuously recorded using two auxiliary actiCAP flat electrodes (Brain Products 2022).

We processed EEG data offline using BrainVision Analyzer (Brain Products 2022). First, we visually determined channels with excessive noise or no signals and conducted topographic interpolation for these channels using quartic spherical splines (Perrin et al. 1989). The mean number of interpolated channels for each participant was 0.89 (SD = 1.09; range: 0–4). For information on interpolation for each electrode, see Table S2. Second, we re‐referenced EEG data to the average of the left and right mastoid electrodes. Third, we bandpass filtered EEG data from 0.1 to 30 Hz using a zero‐phase shift Butterworth filter with roll‐off slope of 12 dB/octave. Fourth, to analyze neural activity during the period of silence between the caller's request and the recipient's affirmative response, we segmented EEG data from −500 to 1500 ms relative to the onset of the inter‐speaker gap in individual trials; to analyze neural activity elicited by the recipient's affirmative response, we segmented EEG data from −200 to 1000 ms relative to the onset of the affirmative “sure” response in individual trials. Fifth, we corrected for ocular artifacts using the algorithm developed by Gratton et al. (1983). Sixth, we conducted artifact rejection using a semi‐automatic procedure on individual channels and segments. Specifically, EEG data spanning 200 ms before and after the occurrence of any of the following conditions were identified as artifacts: (a) rate of change of voltage between two consecutive data points was greater than 50 μV/ms; (b) difference between the minimum and maximum voltages within any 200‐ms interval was greater than 200 μV; or (c) difference between the minimum and maximum voltages within any 100‐ms interval was lesser than 0.5 μV. We visually inspected EEG data to identify additional artifacts. On average, 4.1% (SD = 6.7; range: 0–35.3) of segments were rejected as artifacts for each participant. Seventh, we averaged segments separately for trials with long and normal inter‐speaker gaps. The average number of trials used to calculate the P3 was 9.813 (SD = 0.488) for normal inter‐speaker gaps and 9.775 (SD = 0.699) for long inter‐speaker gaps. The average number of trials used to calculate the SPN was 9.513 (SD = 0.879) for normal inter‐speaker gaps and 9.550 (SD = 0.955) for long inter‐speaker gaps. For information on the individual electrodes included in analysis, see Table S1. We individually inspected subject‐level averages for EEG waveform quality but did not have an official minimal threshold for inclusion. Finally, we performed baseline correction on the averaged EEG data with respect to the interval between −200 and −100 ms before the onset of the inter‐speaker gap or affirmative “sure” response.

For trials with long inter‐speaker gaps, we scored the SPN as the mean amplitude from 200 to 700 ms after the onset of the inter‐speaker gap across electrodes Fz, F3, and FC1. We scored the P3 complex as the mean amplitude from 200 to 400 ms after the onset of the affirmative “sure” response across electrodes Fz, FC1, FC2, and Cz. The analyzed electrodes for the SPN and P3 were selected based on visual inspection of the maximal brain activity within subjects blind to any correlations across subjects. Additionally, based on Foti and Roberts (2016), we expected the SPN would be maximal at frontal left lateralized sites and that the P3 would be maximal at fronto‐central sites. Thus, we intended to balance consistency with past scoring of the SPN and P3 on this task with the topography of the current sample. The P3 complex was scored separately for trials with long and normal inter‐speaker gaps. Additionally, we computed the difference in P3 complexes between trials with long and normal inter‐speaker gaps.

### 2.7 Analytic Strategy

We first conducted descriptive analyses of the BAP and cognitive assessment measures. Specifically, we determined raw scores and Cronbach's alphas for BAPQ total and subscale scores to characterize participants' autism‐associated traits. We interpreted Cronbach's alphas as poor (≤ 0.50), moderate (0.51–0.75), good (0.76–0.90), or excellent (≥ 0.91; Portney 2020). We also determined standard scores for the NIHTB‐CB and language‐focused subtests to characterize participants' cognitive and language abilities. For our first aim of replicating the findings of Foti and Roberts (2016) in the BAP, we examined within‐subject biobehavioral effects of inter‐speaker gap duration. We used the one‐sample *t*‐test to analyze the SPN elicited during trials with long inter‐speaker gaps (i.e., SPN_long_) and two separate paired‐samples *t*‐tests to contrast P3 complexes and ratings elicited by long versus normal inter‐speaker gaps (i.e., ΔP3 = P3_long_ − P3_normal_ and ΔRating = Rating_long_ − Rating_normal_, respectively). For our second aim of investigating associations between the BAP and processing of inter‐speaker gaps, we conducted between‐subjects analyses by computing bivariate correlations between BAPQ total score and each of the three biobehavioral effects (i.e., SPN_long_, ΔP3, and ΔRating). For our third aim of exploring specific BAP features that may be driving overall associations between the BAP and biobehavioral effects, we conducted subscale analyses by computing bivariate correlations between BAPQ subscale scores and biobehavioral effects that were significantly associated with BAPQ total score. Additionally, we quantified effect sizes using Cohen's *d* and Pearson's *r* for all statistical analyses.

## 3 Results

### 3.1 Descriptive Analyses

#### 3.1.1 Autism‐Associated Traits

Table 2 summarizes raw scores and internal consistencies for the BAPQ and its subscales. Substantial variability was observed across BAPQ total and subscale scores, suggesting that our oversampling strategy was successful in yielding a sample of individuals with a broad continuum of autism‐associated traits (see Figure S1 for the full distribution of BAPQ total score). The variability we saw was comparable to more targeted samples in non‐college populations, such as relatives of those diagnosed with ASD (Camodeca and Voelker 2016; O'Connor et al. 2025). In our sample, the BAPQ exhibited strong psychometric properties with good to excellent internal consistencies across total and subscale scores. Notably, male (*M* = 3.10, SD = 0.91) and female (*M* = 3.18, SD = 0.91) participants did not differ in BAPQ total score, *t*(61) = 0.34, *p* = 0.736, *d* = 0.09, 95% CI [−0.38, 0.54].^4^

**TABLE 2:** Raw scores and internal consistencies of Broad Autism Phenotype Questionnaire.

Scale/Subscale (scoring range)	Raw score			*α*
	*M*	SD	Range	
Broad Autism Phenotype Questionnaire (1–6)	3.14	0.90	1.50–5.22	0.96
Aloof personality (1–6)	3.15	1.22	1.00–5.42	0.96
Pragmatic language problems (1–6)	2.96	0.81	1.58–4.83	0.85
Rigid personality (1–6)	3.31	1.04	1.58–5.83	0.91

#### 3.1.2 Cognitive and Language Functioning

Our sample consisted of adults with high cognitive and language abilities. Participants obtained a mean total composite score of 115.79 (SD = 11.46; range: 91–138) on the NIHTB‐CB, corresponding to the high‐average range. As a whole, participants' cognitive functioning was greater than the normative score of 100, *t*(62) = 10.94, *p* < 0.001, *d* = 1.38, 95% CI [112.91, 118.68]. Additionally, participants demonstrated strong expressive and receptive language skills, as evidenced by mean standard scores of 124.06 (SD = 16.15; range: 95–146) and 111.97 (SD = 14.35; range: 76–137) on the Oral Reading Recognition Test and Picture Vocabulary Test, respectively.

### 3.2 Biobehavioral Markers of Conversational Inter‐Speaker Gaps

#### 3.2.1 Preliminary Analyses

**FIGURE 1:**
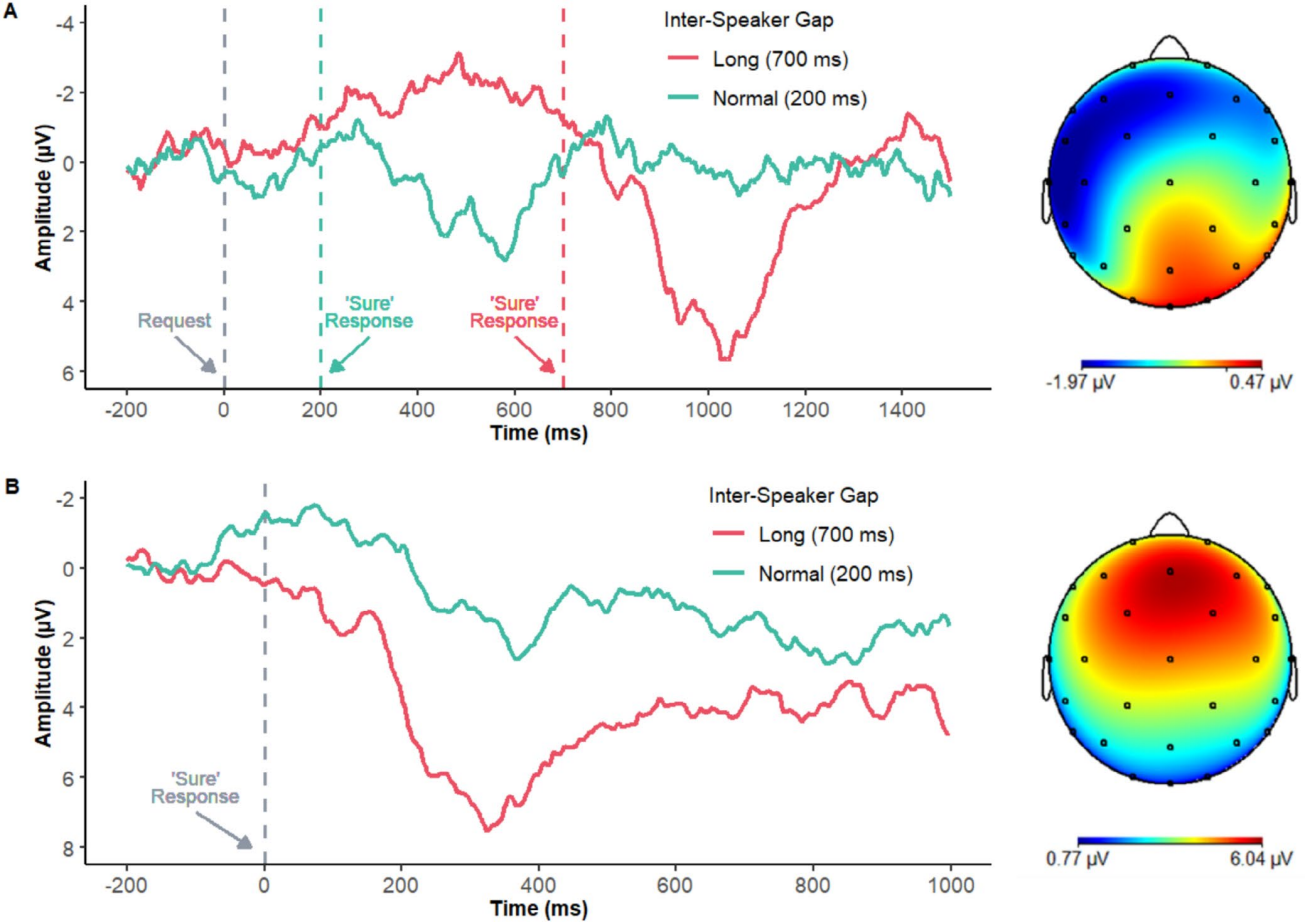
Grand‐average event‐related potentials and scalp topographic maps by inter‐speaker gap. Grand‐average waveforms for (A) stimulus‐preceding negativity (SPN) at electrode Fz and (B) P3 complex at electrode Cz are shown separately for trials with long (700 ms; red line) and normal (200 ms; green line) inter‐speaker gaps between requests and affirmative “sure” responses. The SPN is time‐locked to the onset of the inter‐speaker gap; the scalp topographic map depicts neural activity during the 200–700 ms time window for trials with long inter‐speaker gaps (i.e., SPN_long_). The P3 complex is time‐locked to the onset of the affirmative “sure” response; the scalp topographic map depicts the difference in neural activity during the 200–400 ms time window between trials with long and normal inter‐speaker gaps (i.e., ΔP3).

**FIGURE 2:**
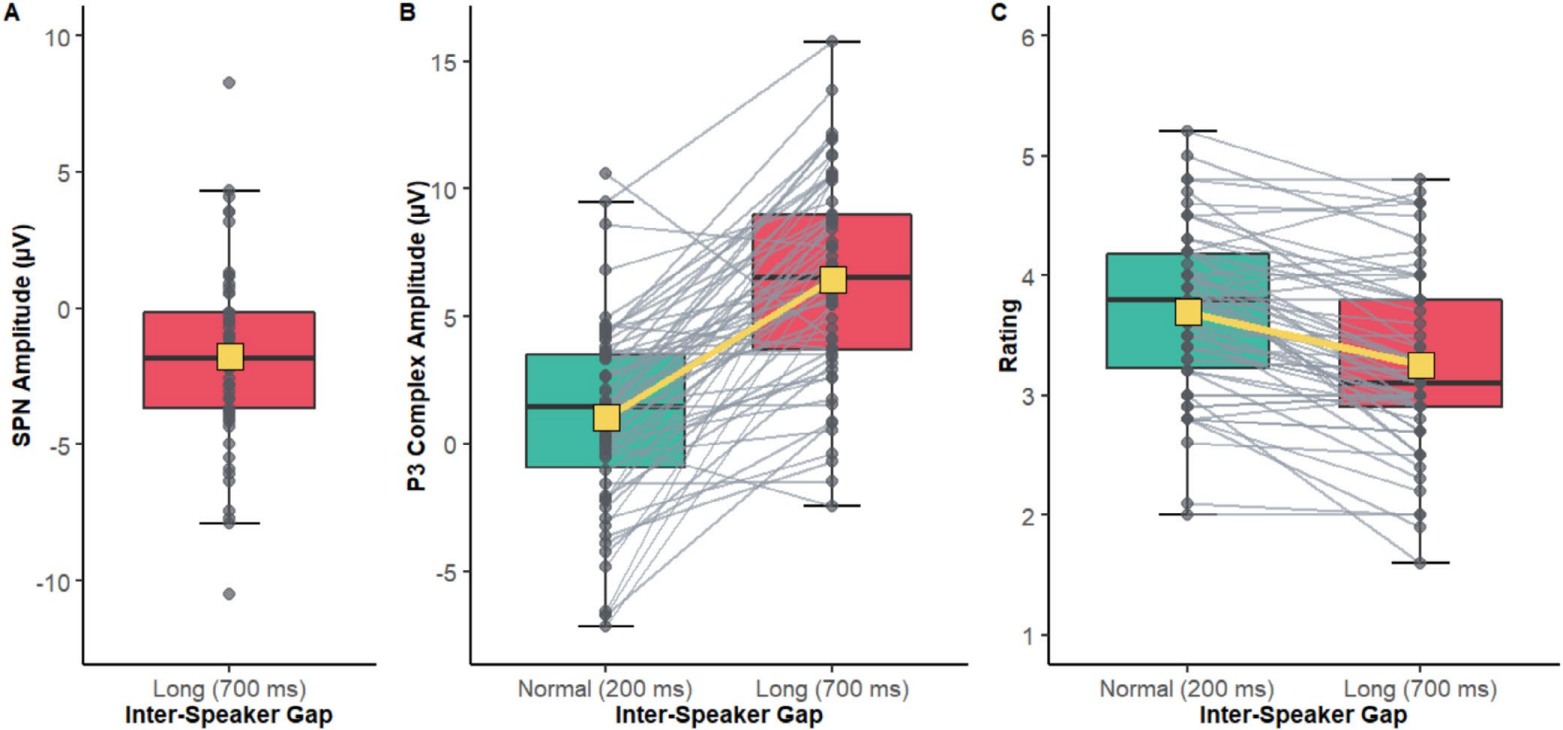
Biobehavioral responses by inter‐speaker gap. Biobehavioral responses, including (A) stimulus‐preceding negativity amplitude, (B) P3 complex amplitude, and (C) rating, are shown separately for trials with long (700 ms) and normal (200 ms) inter‐speaker gaps between requests and affirmative responses. Gray circles represent individual participant data and yellow squares represent mean values.

Preliminary analyses of the three biobehavioral effects of inter‐speaker gap duration (i.e., SPN_long_, ΔP3, and ΔRating) identified a minimal number of outliers, defined as values exceeding three times the interquartile range below the first quartile or above the third quartile, which were excluded from subsequent analyses.^5^ Figure 1 illustrates grand‐average ERP waveforms of the SPN and P3 complex for trials with long and normal inter‐speaker gaps as well as scalp topographic maps for SPN_long_ and ΔP3. Figure 2 depicts individual variations in biobehavioral effects as well as contrasts within‐subject differences in P3 complexes and ratings between trials with long and normal inter‐speaker gaps.

#### 3.2.2 Aim 1: Within‐Subject Analyses of Event‐Related Potential and Behavioral Measures

Broadly, we replicated biobehavioral effects of inter‐speaker gap duration identified by Foti and Roberts (2016). As expected, the SPN was sustained throughout long inter‐speaker gaps, with a mean amplitude of −1.77 μV (SD = 3.23) that was significantly less than zero, *t*(60) = −4.28, *p* < 0.001, *d* = −0.55, 95% CI [−2.60, −0.94], indicating that participants were monitoring and anticipating the affirmative responses to requests. The split‐half reliability for the SPN on long inter‐speaker gaps was inadequate (0.307). Consistent with our predictions, long inter‐speaker gaps (*M* = 6.45 μV, SD = 3.97) elicited larger P3 complexes than normal inter‐speaker gaps (*M* = 1.02 μV, SD = 3.75), *t*(61) = 9.58, *p* < 0.001, *d* = 1.22, 95% CI [4.30, 6.56], suggesting that participants allocated greater attention to affirmative responses to requests following prolonged conversational inter‐speaker gaps. For the P3 on normal inter‐speaker gaps, the split‐half reliability was inadequate (0.273), which is to be expected given the minimal P3 response on these trials. P3 reliability was adequate (0.647) on trials with long inter‐speaker gaps, and the reliability was intermediate for the P3 difference score (0.477). Similarly, affirmative responses to requests after long inter‐speaker gaps (*M* = 3.25, SD = 0.73) were rated more negatively than those after normal inter‐speaker gaps (*M* = 3.69, SD = 0.68), *t*(61) = −7.21, *p* < 0.001, *d* = −0.92, 95% CI [−0.56, −0.32], reflecting differential affective perceptions of acoustically identical affirmative responses following long versus normal inter‐speaker gaps.

#### 3.2.3 Aim 2: Between‐Subjects Analyses of Inter‐Speaker Gaps in the Broad Autism Phenotype

**FIGURE 3:**
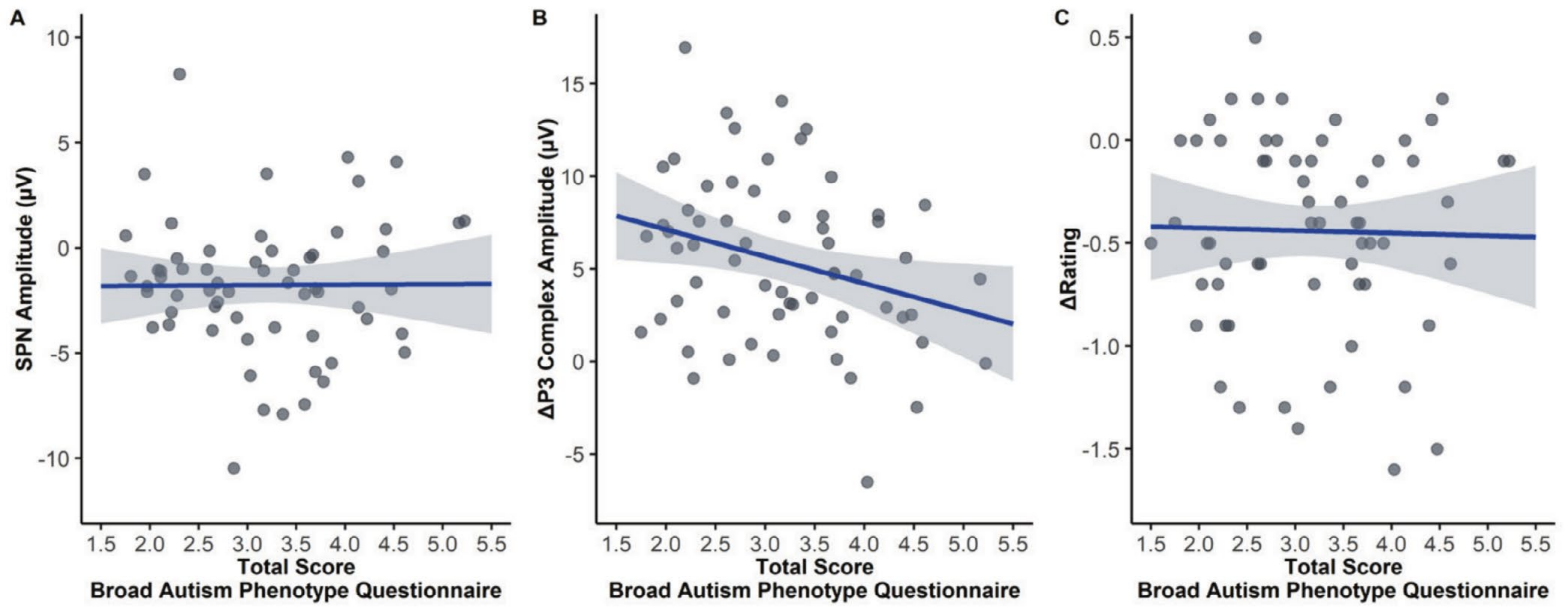
Scatterplots of broad autism phenotype questionnaire total score and biobehavioral markers of conversational inter‐speaker gaps. Associations between Broad Autism Phenotype Questionnaire (BAPQ) total score and (A) stimulus‐preceding negativity elicited by long inter‐speaker gap (i.e., SPN_long_), (B) difference in P3 complex amplitudes elicited by long and normal inter‐speaker gaps (i.e., ΔP3), and (C) difference in ratings elicited by long and normal inter‐speaker gaps (i.e., ΔRating) are depicted as scatterplots. Only BAPQ total score and ΔP3 had a significant negative association.

Table 3 and Figure 3 present bivariate associations between BAPQ total score and biobehavioral effects of inter‐speaker gap duration. BAPQ total score was not significantly correlated with SPN_long_, suggesting that the monitoring of inter‐speaker gaps and anticipation of delayed responses to requests may be unaffected in the BAP. On the contrary, BAPQ total score was negatively associated with ΔP3, such that individuals with more autism‐associated traits exhibited smaller differences in P3 complex amplitudes between trials with long and normal inter‐speaker gaps. A nonsignificant association between BAPQ total score and ΔRating was observed, indicating that participants, regardless of their degree of autism‐associated traits, evaluated affirmative responses following long inter‐speaker gaps more negatively than those following normal inter‐speaker gaps. Of note, the relation between ΔP3 and ΔRating was non‐significant (*r* = −0.050, *p* = 0.699).

**TABLE 3:** Bivariate associations between Broad Autism Phenotype Questionnaire and biobehavioral markers of conversational inter‐speaker gaps.

Scale/Subscale	SPN_long_			ΔP3			ΔRating		
	*r*	*p*	95% CI	*r*	*p*	95% CI	*r*	*p*	95% CI
BAP Questionnaire	0.01	0.961	[−0.25, 0.26]	−0.29	**0.023**	[−0.50, −0.04]	−0.02	0.858	[−0.27, 0.23]
Aloof personality				−0.28	**0.030**	[−0.49, −0.03]			
Pragmatic language				−0.22	*0.084*	[−0.44, 0.03]			
Rigid personality				−0.25	**0.049**	[−0.47, 0.00]			

*Note:* Significant (*p* < 0.05) and marginal (*p* < 0.10) effects are shown in bold and italics, respectively. For any biobehavioral effect, associations at the subscale level were only analyzed as follow‐up tests in the event of a significant association at the scale level (i.e., total score). SPN_long_ = stimulus‐preceding negativity elicited by long inter‐speaker gap; ΔP3 = difference in P3 complexes elicited by long and normal inter‐speaker gaps; ΔRating = difference in ratings elicited by long and normal inter‐speaker gaps.

Overall, our between‐subjects analyses provide some evidence that reduced attention to ambiguous responses to requests (e.g., affirmative responses after long inter‐speaker gaps) may be a specific aspect of conversation processing implicated in the BAP, as evidenced by the significant association between BAPQ total score and ΔP3. Notably, the strength of this association remained unchanged, even after controlling for age, sex, cognitive ability (i.e., total composite score on the NIHTB‐CB), and language skills (i.e., standard scores on the Oral Reading Recognition Test and Picture Vocabulary Test). Specifically, the partial correlation between BAPQ total score and ΔP3 was similar to its zero‐order correlation, *r*_partial_ = −0.31, *p* = 0.019, 95% CI [−0.53, −0.05], indicating that the observed negative association between BAPQ total score and ΔP3 remains robust after accounting for demographic and cognitive characteristics. Finally, given that ΔP3 was calculated as a difference score (long gap—normal gap), we also calculated a regression model whereby the P3 responses to normal and long gaps were entered as simultaneous predictors of BAPQ total score. The unique effect of the P3 to long gaps was significant, controlling for the effect of P3 to normal gaps (*β* = −0.273, *p* = 0.043; also see Table S3).

#### 3.2.4 Aim 3: Follow‐Up Analyses of Broad Autism Phenotype Questionnaire Subscales

Given the significant association between BAPQ total score and ΔP3, we further probed and reported the associations between BAPQ subscale scores and ΔP3 in Table 3. Both *aloof personality* and *rigid personality* subscale scores were negatively associated with ΔP3. Interestingly, the bivariate correlation between the *pragmatic language problems* subscale score and ΔP3 was only marginally significant. These patterns of findings suggest that the overall association between BAPQ total score and ΔP3 may not have been driven by specific BAP features, as assessed by BAPQ subscales. Substantial correlations among BAPQ subscales may have also made it challenging to obtain differential associations between ΔP3 and each subscale (*r*_Aloof/Pragmatic_ = 0.68, *p* < 0.001; *r*_Aloof/Rigid_ = 0.66, *p* < 0.001; *r*_Pragmatic/Rigid_ = 0.64, *p* < 0.001).

## 4 Discussion

Pragmatic language differences are well documented in ASD and the BAP, though the neural underpinnings of specific social language processes have received limited attention. Prior studies have begun to elucidate neural mechanisms involved in different stages of conversation processing (van Berkum 2012), including how different inter‐speaker gap durations may elicit differential neural responses and behavioral ratings (Foti and Roberts 2016). To our knowledge, the present study is the first to examine biobehavioral effects of inter‐speaker gap duration in relation to the BAP using an established conversational task, serving as a critical intermediate step in extending studies involving neurotypical individuals to those with ASD and laying the foundation for potential clinical applications, such as targeted interventions for specific conversational skills.

The present study yielded three major findings. First, using within‐subject analyses, we replicated all biobehavioral effects of inter‐speaker gap duration previously demonstrated in an unselected adult sample (Foti and Roberts 2016). On average, participants in the present study who varied in their degree of autism‐associated traits exhibited a sustained SPN during long inter‐speaker gaps; participants' neural and behavioral responses were also sensitive to the manipulation of inter‐speaker gap duration, with long inter‐speaker gaps resulting in larger P3 complexes and more negative ratings than normal inter‐speaker gaps. Broadly, these replicated findings in a sample selected for high and low features of the BAP suggest that within‐subject biobehavioral effects of inter‐speaker gap duration are likely to be robust, warranting additional studies that focus on clinical samples with ASD and other social communication challenges.

Interestingly, these biobehavioral effects have also been reported in a separate body of literature on communication technology. For example, Uhrig et al. (2018) examined how end‐to‐end delay in telephone networks might affect the perceived quality of conversations and found that longer delays in transmission were associated with lower perceived quality; their electrophysiological analyses further revealed that transmission delays significantly modulated oscillatory neural activity in the beta and gamma frequency bands, which they interpreted as reflecting changes in attentional processing and concentration. These parallel findings highlight the possibility that inter‐speaker gaps arising from both natural and technical causes may be similarly processed. Given increasing use of digital technologies (e.g., embodied conversational agents and virtual reality systems) for promoting conversational skills in ASD (Rosenfield et al. 2019), future work may consider using time‐frequency analyses to integrate these findings, which may better capture neural dynamics and developmental changes (Morales and Bowers 2022).

Second, as expected, our between‐subjects analyses demonstrated that differences in P3 complexes between long and normal inter‐speaker gaps were inversely correlated with the degree of autism‐associated traits. This suggests that some features of the BAP may be associated with reduced sensitivity to uncharacteristically extended inter‐speaker gaps during conversations. Notably, this association remained significant after accounting for demographic factors and broad cognitive and language abilities. In contrast, the SPN during long inter‐speaker gaps was independent of the degree of autism‐associated traits, providing some support that the monitoring of inter‐speaker gaps—even those that are uncharacteristically extended—may be unaffected in relation to the BAP. Collectively, these findings suggest that affirmative responses to requests after long inter‐speaker gaps may be perceived as less salient for individuals with more autism‐associated traits, offering preliminary evidence that allocation of attention to ambiguous verbal responses may be a specific aspect of conversation processing that is relevant to the BAP. Our covariate analyses further suggest that broad differences in cognitive and language functioning are unlikely explanations for the patterns of results that we observed. Given that the two ERPs (i.e., SPN and P3) have been shown to map onto dissociable neural networks (Foti and Roberts 2016), it is plausible that the degree of autism‐associated traits may be related to neuroanatomical and functional differences in the anterior pathway of language processing, which is responsible for decoding word content in conversations, but not in the posterior pathway, which is involved in monitoring conversational turn‐taking (Scott et al. 2009). Indeed, neural structures involved in the anterior pathway include the superior temporal gyrus and medial prefrontal cortex (Foti and Roberts 2016), both of which have been found to be weakly activated when autistic individuals perform theory‐of‐mind tasks (Frith 2003). Future multimodal neuroimaging studies with more targeted BAP and ASD samples may lead to a better understanding of neural networks that are involved in monitoring and interpreting conversational inter‐speaker gaps.

Finally, no specific BAP characteristics seemed to be driving the overall association between the degree of autism‐associated traits and differences in P3 complexes elicited by long versus normal inter‐speaker gaps, as evidenced by at least marginally significant associations across all BAPQ subscales in our follow‐up analyses. The substantial correlations between pairs of subscales may partially account for the lack of differential findings across subscales, with these subscale correlations having similar magnitudes to those reported during the development of the BAPQ (Hurley et al. 2007). Additionally, given that the overall association plausibly reflects a specific aspect of conversation processing (i.e., pragmatic inference of inter‐speaker gaps), it is likely that only a subset of the heterogenous items within a subscale have a direct correspondence to this specific biobehavioral effect. For example, the *pragmatic language problems* subscale included items that appear to be more closely related to understanding pragmatic aspects of conversations (e.g., “I feel disconnected or “out of sync” in conversations with others” and “I can tell when someone is not interested in what I am saying”) as well as items that likely measure broader social communication constructs (e.g., “I find it hard to get my words out smoothly” and “I speak too loudly or softly”). To further explore specific BAP features that may be especially relevant for processing conversational inter‐speaker gaps, additional studies that leverage item‐level analyses in larger samples of individuals with varied BAP characteristics will be invaluable. Additionally, utilizing behavioral assessments of pragmatic language may be particularly useful to clarify this relation. Performance‐based tests, such as the Test Of Pragmatic Language‐2 (TOPL‐2; Phelps‐Terasaki and Phelsp‐Gunn 2007), and behavioral coding of pragmatic skills, such as with the Pragmatic Rating Scale (PRS; Landa et al. 1992), have been used to directly assess pragmatic language (Klusek et al. 2016; Drumm et al. 2015; Losh et al. 2012).

Consistent with prior research using this EEG task (Foti and Roberts 2016), a negative‐going slow wave was observed at left frontocentral electrodes during the inter‐speaker gap. This slow wave is functionally consistent with the SPN, given that it was elicited during the anticipation of the auditory stimulus conveying speaker response. It should be noted that the scalp topography of the SPN varies depending on the nature and modality of the anticipated stimulus. For example, when anticipating performance‐based feedback, the SPN typically has a centroparietal scalp distribution (Foti and Hajcak 2012; Novak et al. 2016), whereas the anticipation of affective stimuli elicits a frontally‐maximal SPN (Takeuchi et al. 2005). With regard to modality, the anticipation of visual stimuli elicits an occipital SPN whereas auditory stimuli elicit a frontal SPN (Brunia and van Boxtel 2004). With these factors in mind, the observed frontal SPN here is to be expected given that on this task participants were anticipating an auditory stimulus conveying a speaker response that shaped the perceived affective tone of the conversation. It is also possible, however, that this frontocentral slow wave may partially reflect motor preparation consistent with the contingent negative variation (CNV) given that participants made a button press following the speaker response. The relative contributions of the SPN versus CNV to the observed slow wave could be disentangled in future research by including trials with and without a behavioral response.

Relatedly, the observed P3 complex here was maximal at frontocentral electrodes. This is more consistent with the canonical P3a rather than the P3b, which is typically maximal at parietal electrodes. The P3a is thought to index the allocation of attention to salient stimuli, whereas the subsequent P3b is more closely linked to stimulus categorization and decision‐making (Barry et al. 2016). Here, the affirmative speaker responses were identical auditory stimuli. It is plausible that a more complex task design with varying speaker responses—thus requiring more effortful stimulus categorization—might also modulate the P3b.

Findings from the present study must be considered in the context of a few limitations, including the size and nature of our sample and the scope of pragmatic features that we examined. While our sample of 63 participants was sufficient for detecting medium effect sizes, it is possible that some of our null findings (e.g., non‐significant association between BAPQ total score and SPN during long inter‐speaker gaps) may be due to low statistical power for uncovering smaller effect sizes. Similarly, although our convenience sampling approach yielded individuals with a broad continuum of autism‐associated traits, our sample of university students may not fully represent the BAP in the general population and may give rise to some concerns commonly raised in psychological research involving student samples (e.g., generalizability; Henrich et al. 2010). Further research using larger and more diverse samples is therefore warranted to replicate and extend our findings.

With regard to EEG task design, we were not able to fully disentangle the processing of inter‐speaker turn‐taking from basic sensory processing, which is relevant given work linking autism to reduced auditory gap detection and broader temporal binding windows (Foss‐Feig et al. 2018; Zhou et al. 2018). Future studies could examine this further by including a more comprehensive EEG battery of tasks that look at both conversation processing and sensory processing. Further, the relatively small number of trials limited the reliability of the observed ERPs. Future studies may consider an expanded task with more trials, which should improve reliability and the ability to detect associations with BAP and other individual difference factors.

Additionally, the present study focused on inter‐speaker gaps, a key pragmatic feature in conversations that has been well characterized in cross‐linguistic and empirical studies on pragmatic language (Levinson and Torreira 2015; Roberts and Francis 2013; Stivers et al. 2009). Nevertheless, conversation processing is complex and involves several verbal and nonverbal pragmatic components (e.g., intra‐speaker pauses, prosodic attributes, gaze patterns, and gestures; Sidnell and Stivers 2013), necessitating additional studies to comprehensively understand the range of pragmatic features that may be implicated in the BAP and ASD. Future work may consider adopting the psychophysiological approach used in the present study, which may yield insights on shared and distinct neural bases for processing individual pragmatic features in conversations. In the present study, we leveraged an established conversational task that constrained inter‐speaker gap duration to either 200 or 700 ms. Given that real‐world inter‐speaker gap durations are more continuous in nature, it may be beneficial to include an expanded set of inter‐speaker gap durations in future studies to enhance ecological validity. Notably, operationalizing inter‐speaker gap duration as a continuous metric may hold clinical value, where nuanced changes in biobehavioral sensitivity to inter‐speaker gaps may be used to monitor progress in clinical interventions.

In conclusion, the present study builds on the cognitive neuroscience literature on pragmatic language by examining processing of conversational inter‐speaker gaps in the BAP. Using a sample of individuals specifically selected to vary in their degree of autism‐associated traits, we investigated biobehavioral effects of appropriate versus uncharacteristically extended inter‐speaker gap durations. We demonstrated that pragmatic inference of inter‐speaker gaps—as indicated by a larger P3 complex amplitude to speaker response following an atypically long inter‐speaker gap—may vary with features of the BAP consistent with broader known pragmatic language differences in the BAP. Extending this research approach to clinical populations, including ASD, may advance our theoretical understanding of neural processes that underlie conversation processing and inform clinical approaches for promoting effective social communication skills.

## Author Contributions

**Wei Siong Neo:** writing – original draft, formal analysis, project administration, methodology, visualization, investigation. **Kamryn M. Witkowiak:** writing – review and editing, formal analysis, visualization. **Dan Foti:** conceptualization, writing – review and editing, supervision, methodology. **Mai Yamamoto:** project administration, resources. **Felicia Roberts:** conceptualization, funding acquisition, supervision, methodology. **Bridgette Kelleher:** conceptualization, supervision, methodology.

## Funding

This work was supported by funding from the Brian Lamb School of Communication, Purdue University.

## Ethics Statement

This research was formally approved by the IRB at Purdue University. All participants provided written informed consent.

## Conflicts of Interest

The authors declare no conflicts of interest.

## Supporting information


**Table S1:** Mean trials used to calculate the ERP average for each electrode.
**Table S2:** Number of participants each electrode was interpolated for.
**Table S3:** Results of a multiple linear regression of P3_normal_ and P3_long_ onto total BAPQ score.
**Table S4:** Raw scores and internal consistencies of supplemental broad autism phenotype measures.
**Table S5:** Bivariate associations between supplemental broad autism phenotype measures and biobehavioral markers of conversational inter‐speaker gaps.
**Figure S1:** Total score distributions of broad autism phenotype measures.

## Data Availability

The data that support the findings of this study are available from the corresponding author upon reasonable request.

## Endnotes

^1^
The prescreening questionnaire included questions 1, 3, 9, 11, 14, 15, 16, 17, 19, 26, and 36 from the BAPQ.

^2^
Participants also completed two other measures frequently used in the BAP literature: Autism‐Spectrum Quotient (AQ; Baron‐Cohen et al. 2001) and Social Responsiveness Scale, Second Edition (SRS‐2; Constantino and Gruber 2012). We focused on the BAPQ because it was specifically developed to assess the BAP and exhibits superior psychometric properties than the AQ and SRS‐2 (Ingersoll et al. 2011). Parallel analyses for the AQ and SRS‐2 are reported in the Supporting Information.

^3^
A sample conversation between the caller (C) and the recipient (R) on a target trial is: [ring ring] (R) Hello. (C) Rachel? (R) Yeah. (C) Hey, it is me. (R) Hey, how is it going? (C) Good. I just talked to Office World. (R) Uh‐huh. (C) And the desk is ready. Can you give me a ride over there? (R) Sure. A sample conversation on a distractor trial is: [ring ring] (R) Hello. (C) Hey, how is it going? (R) Good. What is going on? (C) Not much, but I just heard that Adam is coming up for the weekend. (R) Oh great.

^4^
While male and female participants did not differ in BAPQ total score and subscale scores (*aloof personality*: *t*(61) = 1.17, *p* = 0.248, *d* = 0.29, 95% CI [−0.26, 0.97]; *pragmatic language problems*: *t*(61) = −0.66, *p* = 0.511, *d* = −0.17, 95% CI [−0.55, 0.28]; *rigid personality*: *t*(61) = 0.04, *p* = 0.966, *d* = 0.01, 95% CI [−0.52, 0.54]), a growing body of literature indicates that males and females with ASD present with distinctive behavioral symptoms, which may be underpinned by sex‐specific biobehavioral mechanisms (Ferri et al. 2018; Kreiser and White 2014). It is plausible that such sex differences extend to the BAP.

^5^
We identified and excluded two SPN_long_ outliers (−15.42 and 10.98 μV), one △P3 outlier (−15.14 μV), and one △Rating outlier (−2.7).
