# Supplementary material for: Neural Sensitivity to Conversational Inter‐Speaker Gaps in the Broad Autism Phenotype
Source: Psychophysiology. 2026 Jul 3;63(7):e70355. doi: 10.1111/psyp.70355 (PMC13332344; doi:10.1111/psyp.70355)
Supplement: Supplementary file 1 — Table S1: Mean trials used to calculate the ERP average for each electrode. Table S2: Number of participants each electrode was interpolated for. Table S3: Results of a multiple linear regression of P3normal and P3long onto total BAPQ score. Table S4: Raw scores and internal consistencies of supplemental broad autism phenotype measures. Table S5: Bivariate associations between supplemental broad autism phenotype measures and biobehavioral markers of conversational inter‐speaker gaps. Figure S1: Total score distributions of broad autism phenotype measures. [file PSYP-63-e70355-s001.docx]

**Supplemental Materials**

**Supplemental Broad Autism Phenotype Measures**

In addition to the Broad Autism Phenotype Questionnaire (BAPQ), participants also completed two supplemental broad autism phenotype (BAP) measures: Autism-Spectrum Quotient (AQ) and Social Responsiveness Scale, Second Edition (SRS-2). We conducted supplemental analyses for the AQ and SRS-2 in accordance with the analytic approach that we used in primary analyses for the BAPQ.

***Descriptive Analyses***

Supplemental Table 4 summarizes raw scores and internal consistencies for both AQ and SRS-2, and Supplementary Figure 1 depicts their total score distributions. Similar to the BAPQ, substantial variability was observed across AQ and SRS-2 total and subscale scores. Internal consistencies were good to excellent for SRS-2 total score and all its subscales, except for the *social awareness* subscale that had poor internal consistency. In contrast, while the AQ total score had good internal consistency, moderate internal consistencies were obtained for all its subscales. Similar to the BAPQ, male and female participants did not differ in AQ total score (*M*_male_ = 21.94, *SD*_male_ = 7.70; *M*_female_ = 22.56, *SD*_female_ = 8.54; *t*(61) = 0.31, *p* = .761, *d* = 0.08, 95% CI [−3.47, 4.73]) and SRS-2 total score (*M*_male_ = 61.26, *SD*_male_ = 32.03; *M*_female_ = 65.50, *SD*_female_ = 33.54; *t*(61) = 0.51, *p* = .610, *d* = 0.13, 95% CI [−12.29, 20.77]).

***Between-Subjects Analyses***

Supplemental Table 5 presents bivariate associations between total scores of both supplemental BAP measures and biobehavioral effects of inter-speaker gap duration. Similar to the BAPQ, both AQ and SRS-2 total scores were not significantly correlated with SPN_long_. While BAPQ total score was negatively associated with ΔP3, the negative associations between both AQ and SRS-2 total scores and ΔP3 were not statistically significant. Similar to the BAPQ, both AQ and SRS-2 total scores were not significantly correlated with ΔRating.

**Supplemental Table 1**

*Mean Trials Used to Calculate the ERP Average for each Electrode*

|  | Fz | F3 | FC1 | FC2 | Cz | Total |
| --- | --- | --- | --- | --- | --- | --- |
| P3 Normal | 9.73 | 9.72 | 9.80 | 9.83 | 9.81 | 9.78 |
| P3 Long | 9.67 | 9.77 | 9.81 | 9.8 | 9.84 | 9.78 |
| SPN Normal | 9.34 | 9.45 | 9.55 | 9.59 | 9.63 | 9.51 |
| SPN Long | 9.39 | 9.48 | 9.63 | 9.63 | 9.63 | 9.55 |

*Note.* Electrodes Fz, F3, and FC1 were averaged to calculate the SPN. Electrodes Fz, FC1, FC2, and Cz were averaged to calculate the P3.

**Supplemental Table 2**

*Number of Participants each Electrode was Interpolated for*

| Electrode | Participants Interpolated | % of Sample |
| --- | --- | --- |
| Fp1 | 3 | 4.76 |
| Fz | 0 | 0 |
| F3 | 0 | 0 |
| F7 | 0 | 0 |
| FT9 | 0 | 0 |
| FC5 | 0 | 0 |
| FC1 | 0 | 0 |
| C3 | 0 | 0 |
| T7 | 0 | 0 |
| CP5 | 0 | 0 |
| CP1 | 0 | 0 |
| Pz | 0 | 0 |
| P3 | 0 | 0 |
| P7 | 0 | 0 |
| O1 | 1 | 1.59 |
| Oz | 0 | 0 |
| O2 | 0 | 0 |
| P4 | 0 | 0 |
| P8 | 0 | 0 |
| CP6 | 0 | 0 |
| CP2 | 0 | 0 |
| Cz | 0 | 0 |
| C4 | 0 | 0 |
| T8 | 1 | 1.59 |
| FT10 | 0 | 0 |
| FC6 | 0 | 0 |
| FC2 | 0 | 0 |
| F4 | 0 | 0 |
| F8 | 0 | 0 |
| Fp2 | 6 | 9.5 |

**Supplemental Table 3**

*Results of a Multiple Linear Regression of P3_normal_ and P3_long_ onto Total BAPQ Score*

|  | Unstandardized *B* | *SE* | *β* | *t* | *p* |
| --- | --- | --- | --- | --- | --- |
| P3_normal_ | 0.053 | 0.031 | 0.226 | 1.709 | 0.093 |
| P3_long_ | -0.061 | 0.029 | -0.273 | -2.067 | 0.043 |

**Supplemental Table 4**

*Raw Scores and Internal Consistencies of Supplemental Broad Autism Phenotype Measures*

|  | Raw Score | | |  |
| --- | --- | --- | --- | --- |
| Scale/Subscale (Scoring Range) | *M* | *SD* | Range | α |
| Autism-Spectrum Quotient (0–50) | 22.25 | 8.08 | 9–38 | .86 |
| Attention switching (0–10) | 5.62 | 2.21 | 1–10 | .65 |
| Attention to detail (0–10) | 6.21 | 2.38 | 1–10 | .70 |
| Communication (0–10) | 3.32 | 2.33 | 0–8 | .68 |
| Imagination (0–10) | 3.21 | 1.95 | 0–7 | .53 |
| Social skills (0–10) | 3.90 | 2.69 | 1–10 | .75 |
| Social Responsiveness Scale (0–195) | 63.41 | 32.61 | 19–140 | .96 |
| Social awareness (0–24) | 7.78 | 2.84 | 1–15 | .35 |
| Social cognition (0–36) | 9.86 | 5.42 | 1–23 | .77 |
| Social communication (0–66) | 21.56 | 12.28 | 6–49 | .92 |
| Social motivation (0–33) | 13.59 | 7.93 | 0–29 | .91 |
| Social communication and interaction (0–159) | 52.78 | 25.97 | 16–111 | .95 |
| Restricted interests and repetitive behavior (0–36) | 10.63 | 7.70 | 1–31 | .91 |

**Supplemental Table 5**

*Bivariate Associations between Supplemental Broad Autism Phenotype Measures and Biobehavioral Markers of Conversational Inter-Speaker Gaps*

|  | SPN_long_ | | |  | ΔP3 | | |  | ΔRating | | |
| --- | --- | --- | --- | --- | --- | --- | --- | --- | --- | --- | --- |
| Scale/Subscale | *r* | *p* | 95% CI |  | *r* | *p* | 95% CI |  | *r* | *p* | 95% CI |
| Autism-Spectrum Quotient | −.03 | .842 | [−.28, .23] |  | −.15 | .236 | [−.39, .10] |  | −.06 | .626 | [−.31, .19] |
| Social Responsiveness Scale | .07 | .603 | [−.19, .31] |  | −.21 | .107 | [−.43, .05] |  | .01 | .908 | [−.24, .26] |

*Note*. SPN_long_ = stimulus-preceding negativity elicited by long inter-speaker gap; ΔP3 = difference in P3 complexes elicited by long and normal inter-speaker gaps; ΔRating = difference in ratings elicited by long and normal inter-speaker gaps.

**Supplemental Figure 1**

*Total Score Distributions of Broad Autism Phenotype Measures*


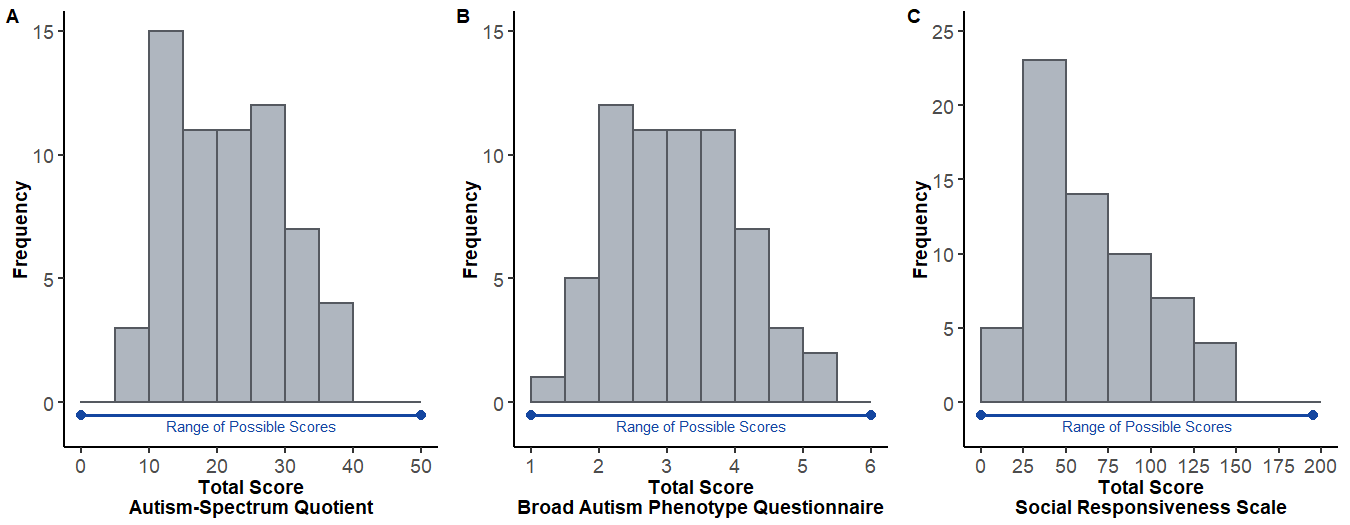


*Note*. Participants’ total scores on the (A) Autism-Spectrum Quotient, (B) Broad Autism Phenotype Questionnaire, and (C) Social Responsiveness Scale are depicted as histograms. The range of possible total scores for each measure is indicated below each corresponding histogram.
